# The Cellular Architectures of Hypospadias

**DOI:** 10.1177/1093526620943084

**Published:** 2020-08-05

**Authors:** Ann Nozohoor Ekmark, Diane Grelaud, Emma Hansson, Henry Svensson, Einar Arnbjörnsson, David Gisselsson

**Affiliations:** 1 Department of Clinical Sciences, Malmö, Lund University, Lund, Sweden; 2 Department of Paediatric Surgery, Skåne University Hospital, Lund, Sweden; 3 Department of Pathology, Skåne University Hospital and Regional Laboratories, Malmö, Sweden; 4 Department of Clinical Sciences, The Sahlgrenska Academy, Gothenburg University, Gothenburg, Sweden; 5 Department of Plastic and Reconstructive Surgery, Sahlgrenska University Hospital, Gothenburg, Sweden; 6 Department of Plastic and Reconstructive Surgery, Skåne University Hospital, Malmö, Sweden; 7 Department of Clinical Sciences Lund, Lund University, Lund, Sweden; 8 Division of Clinical Genetics, Department of Laboratory Medicine, Lund University, Lund, Sweden; 9 Department of Pathology, Skåne University Hospital and Regional Laboratories, Lund, Sweden

Hypospadias is a malformation of the penis characterized by a misformed ventrum, including a superficial urethral plate (UP) and a deep chordee (DC) causing ventral bend. Despite previous histological studies, little is known about the detailed cellular composition of hypospadias.^1–4^ We aimed to characterize the basic histology of the UP and DC in primary hypospadias and make a comparison to secondary repairs.

**Figure 1.**
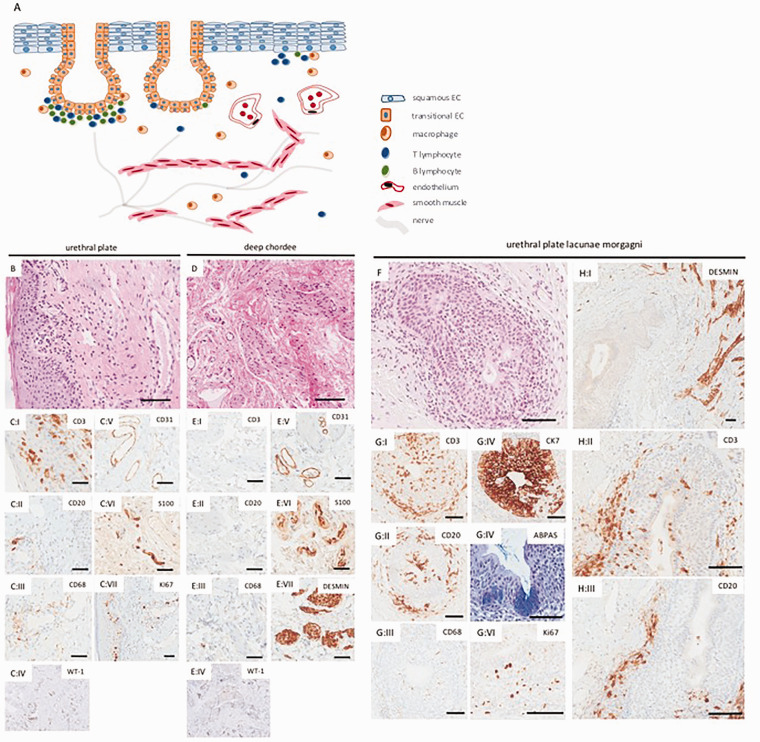
Ideogram summarizing the basic histology of hypospadias (A) followed by representative histology of the urethral plate (B–C) and deep chordee (D–E) from the same patient. B and D represent hematoxylin-eosin staining. Presence of inflammatory cells in the urethral plate is manifested by intraepithelial and subepithelial infiltration of CD3+ T cells (C: I) and sparse subepithelial clusters of CD20+ B cells (C: II) and histiocytes (C: III). There is prominent CD31+ subepithelial vascularization (B and C: V), while S100+ nerve fibers are sparse and slender (C: VI). Proliferation as visualized by Ki67 is confined to the epithelial basal layer and inflammatory cells (C: VII). The deep chordee has fewer inflammatory cells (D and E: I–III). It is dominated by connective tissue with a rich capillary network (E: V), abundant coarse nerve fibers (E: VI) and smoot muscle bundles (E: VII). The endothelium is WT-1 negative in the urethral plate (C: IV) and the deep chordee (E: IV). Urethral plate with lacunae Morgagni (hematoxylin eosin in F) is characterized by moderate lymphocytic infiltrates both in its epithelium and in the surrounding connective tissue (G: I–II, H: II–III), while histiocyte presence is sparse (G: III). The epithelium expresses cytokeratin 7, consistent with a urothelial phenotype (G: IV) and shows focal features of cystitis cystica et glandularis with positivity in alcian blue periodic acid Schiff base staining (G: IV). The proliferating cells fraction was confined to the epithelium and surrounding inflammatory cells. Cysts and canals of the lacunae Morgagni were typically embraced by smooth muscle fibers originating from the direction of the deep chordee (H: I). Scale bars correspond to 50 µm. CD, Clusters of Differentiation.

After approval by the regional ethics review board, penile biopsies were collected from 14 hypospadias patients (1.5–18, median 5 years) and prepared by standard histotechnology. All UP biopsies (n = 9) showed similar characteristics. Underneath the squamous epithelium, ectatic vessels and regional dermal infiltrates of T-cells and B-cells together with sparse macrophages were found (Figure 1(A) and (B)). Focally, T-cells could be seen infiltrating the epithelium (Figure 1 (C:I)). All but one UP had cystic structures with cuboidal urothelium, likely remnants of lacunae Morgagni.^4^ These structures were infiltrated and surrounded by lymphocytes and occasional macrophages (Figure 1(F) and (G:I-VI); Figure 1 (H:I-III)). Subepithelially, there was hypocellular fibrous tissue with moderate to rich vascularity Figure 1 (C:IV-V) with sparse bundles of smooth muscle cells crossing through the stroma along with sparse nerve bundles (Figure 1 (C:VI)). Proliferation was confined to inflammatory cells and the epidermal basal cell layer (Figure 1 (C:VII)).

The DC (n = 13) was characterized by connective tissue with scarce inflammatory cells and fewer vessels than beneath the UP (Figure 1(D) and (E:I-III)). In contrast to the UP, there were prominent bundles of nerves and smooth muscle cells along with irregular vessels of varying media thickness (Figure 1(D) and (E IV-VII)). Vessels and nerves were in some instances parallel, forming a unit. Only 1/5 secondary repairs, an adult with extensive childhood surgery due to an advanced malformation, showed prominent scar tissue, characterized by vast hypocellular areas dominated by collagen bundles. The remaining 4/5 tissues samples from secondary repairs did not show any difference to primary DC samples by our methodological approach.

In summary, our studies indicate that hypospadias has a distinct anatomic pathology, consisting of fibrovascular tissue, covered by squamous epithelium and urothelial pits, where inflammatory cells are a recurrent feature. The methods used in this study could not differentiate between the cellular structure of the DC in primary and secondary cases, possibly due to remaining DC from the original repair. Similar to vascular malformations, the architecture of the arterioles in the DC were slightly irregular and the endothelium wilms tumor protein 1 (WT-1) negative, suggestive of malformed origin rather than vascular proliferation as part of tissue repair.^5^ The inflammatory cells identified in this study have not been previously described in hypospadias,^1–4^ possibly because previous studies did not specifically focus on detecting such cells.^1,^^2,^^4^ The extent to which their presence is a reaction to external cues or an intrinsic component of chordee development remains to be investigated. Our findings raise questions regarding the role of malformed vessels and inflammatory activity in the pathology of hypospadias and its possible impact on its surgical repair.
